# Unraveling Structure‐Dependent Performance of Cu–N–C Molecular Catalysts for Electrocatalytic CO_2_ Methanation

**DOI:** 10.1002/smll.75041

**Published:** 2026-08-02

**Authors:** Tengyi Liu, Xiaofan Hou, Songbo Ye, Kosuke Ishibashi, Yutaro Hirai, Yasutaka Matsuo, Shimpei Ono, Hao Li, Hiroshi Yabu

**Affiliations:** ^1^ Advanced Institute for Materials Research (WPI‐AIMR) Tohoku University Sendai Japan; ^2^ AZUL Energy, Inc. Sendai Japan; ^3^ Research Institute for Electronic Science (RIES) Hokkaido University Sapporo Japan; ^4^ International Center for Synchrotron Radiation Innovation Smart Tohoku University Sendai Japan

**Keywords:** copper phthalocyanine, electrochemical CO_2_ reduction, methane, reaction pathways, structure‐dependent performance

## Abstract

High‐rate CH_4_ electrosynthesis offers a promising strategy for converting CO_2_ into energy‐dense fuels compatible with existing natural gas infrastructure. Cu–N–C molecular catalysts with well‐defined Cu–N_4_ sites show strong potential for CO_2_‐to‐CH_4_ production, yet the role of the macrocyclic backbone remains unclear. Herein, we compare two representative Cu–N–C molecular catalysts, copper phthalocyanine (CuPc) and copper tetraphenylporphyrin (CuPr), to clarify how the macrocyclic backbone influences CO_2_‐to‐CH_4_ electrocatalysis. When supported on conductive carbon and integrated into gas‐diffusion electrodes, CuPc markedly outperforms CuPr, achieving a CH_4_ Faradaic efficiency of 79.5%, a high partial current density of −575 mA cm^−2^, a mass activity of 19 166.7 A g^−1^, and stable operation for over 80 h at −150 mA cm^−2^. Characterization results suggest that the Pc‐macrocyclic backbone modulates the electronic structure of the Cu center and interfacial charge‐transfer behavior, possibly through its extended π‐conjugated Pc‐ring and bridge‐N environment. Theoretical calculations reveal a favorable CH_4_‐forming pathway on the CuPc/C model, with a lower free‐energy barrier for the potential‐determining step than that reported for CuPr, suggesting facilitated CH_4_ formation. These findings indicate that the macrocyclic backbone can regulate reaction pathways and intermediate adsorption energetics, providing mechanistic insight and design guidance for molecular catalysts for CO_2_‐to‐CH_4_ electroreduction.

## Graphical abstract

Copper phthalocyanine (CuPc) and copper tetraphenylporphyrin (CuPr) both contain Cu–N_4_ sites, yet CuPc shows superior CO_2_‐to‐CH_4_ selectivity. Further analysis suggests that the Pc‐macrocycle tunes the electronic structure of Cu center and induces charge transfer, possibly via its π‐conjugated Pc‐ring and bridge‐N environment. DFT calculations indicate a more favorable CH_4_‐forming pathway on CuPc/C, providing mechanistic insight for designing molecular catalysts for CO_2_ methanation.

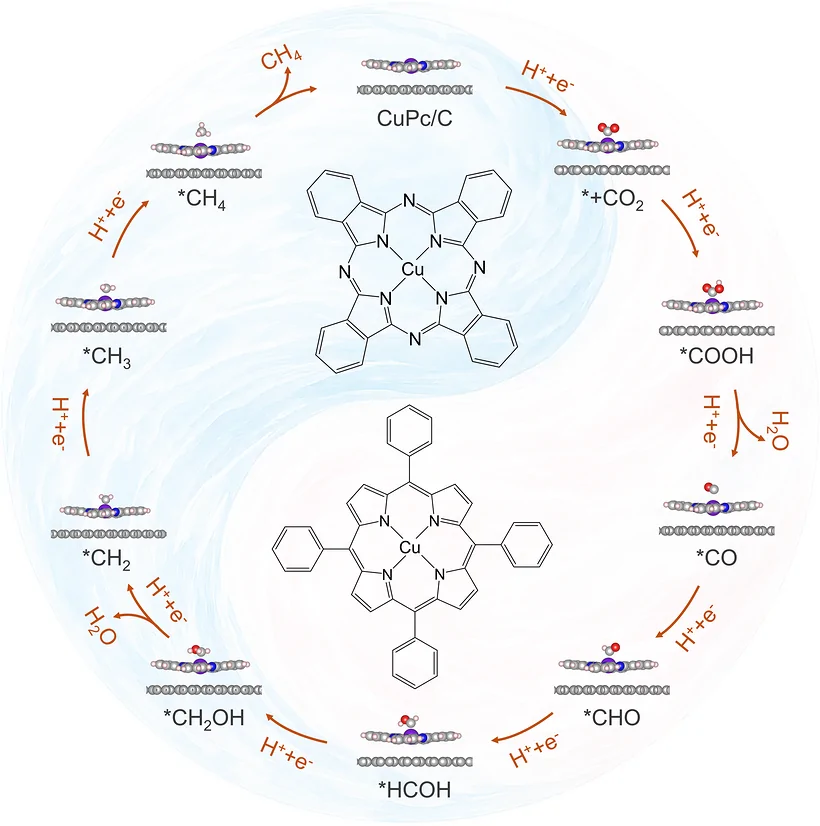

## 1 Introduction

Electrochemical CO_2_ reduction (ECR) provides a mild and sustainable route for CO_2_ utilization, especially when powered by renewable electricity, enabling the conversion of CO_2_ into energy‐dense fuels and value‐added chemicals [1, 2, 3, 4, 5, 6, 7]. Among the various ECR products, methane (CH_4_) has attracted particular attention because of its high energy density and compatibility with existing natural gas infrastructure [8, 9, 10]. As an eight‐electron product, it stores a large number of reducing equivalents, making it an especially attractive target for ECR [11, 12, 13, 14]. However, CO_2_‐to‐CH_4_ conversion involves multiple proton‐coupled electron‐transfer steps and complex reaction intermediates, resulting in sluggish kinetics and numerous competing pathways [11, 15, 16, 17, 18]. Therefore, the development of highly active and selective catalysts remains a major challenge for efficient and selective CH_4_ production in CO_2_ electroreduction.

In recent years, molecular Cu–N–C catalysts with well‐defined coordination structures have shown strong potential for ECR [11, 13, 14, 15, 19, 20]. In particular, phthalocyanine (Pc) and porphyrin‐based molecules have been widely studied because of their stable Cu–N_4_ sites and tunable macrocyclic electronic structures [13, 19, 21]. For example, Zhang and co‐workers improved the catalytic performance of copper tetraphenylporphyrin (CuPr) through peripheral functionalization [19], while Yue and co‐workers reported similar effects for copper phthalocyanine (CuPc) by tuning its peripheral substituents [13]. These studies highlight the importance of molecular structure engineering in Cu–N–C catalysts. However, current studies mainly focus on peripheral modification, and the role of the macrocyclic backbone itself has not been systematically examined.

This question is particularly relevant to two representative Cu–N–C molecular catalysts, CuPc and CuPr. CuPc features a phthalocyanine macrocyclic backbone composed of four isoindole units, whereas CuPr possesses a porphyrin macrocyclic backbone constructed from four pyrrole units with phenyl substituents. Although both catalysts contain Cu–N_4_ active sites, they differ significantly in macrocyclic structure, π‐conjugation, and electronic properties [13, 14, 15, 19]. These differences are likely to influence the electronic state of the Cu center, the adsorption behavior of key intermediates, and the reaction pathway during ECR, thereby affecting catalytic activity and product selectivity [13, 17, 19, 20, 22, 23]. However, the structure–performance relationship between Cu–N–C catalysts with different macrocyclic backbones is still not well understood. Therefore, a direct comparison of CuPc and CuPr can help clarify how the macrocyclic backbone controls CO_2_ reduction.

In this work, CuPc and CuPr were chosen as model catalysts to clarify the role of the macrocyclic backbone in ECR. CuPc exhibits markedly enhanced performance for CH_4_ production, achieving a maximum CH_4_ Faradaic efficiency of 79.5%, a high CH_4_ partial current density of −575 mA cm^−2^, a mass activity of 19 166.7 A g^−1^, and stable operation for over 80 h at −150 mA cm^−2^. These values surpass those of previously reported Pc‐based catalysts. Systematic characterization suggests that the phthalocyanine macrocyclic backbone modulates the electronic structure of the Cu center and the interfacial charge‐transfer behavior, possibly through its extended π‐conjugated Pc ring and bridge‐N environment. Density functional theory (DFT) calculations based on the CuPc/C model further indicate a favorable CO_2_‐to‐CH_4_ pathway with a lower free‐energy barrier for the potential‐determining step than that reported for CuPr. These results suggest that the macrocyclic backbone can regulate intermediate adsorption energetics and the preferred reaction pathway, highlighting the structural advantage of the phthalocyanine framework for CH_4_ formation.

## 2 Results and Discussion

### 2.1 Structural Characterization and Property Comparison of CuPc and CuPr

**FIGURE 1:**
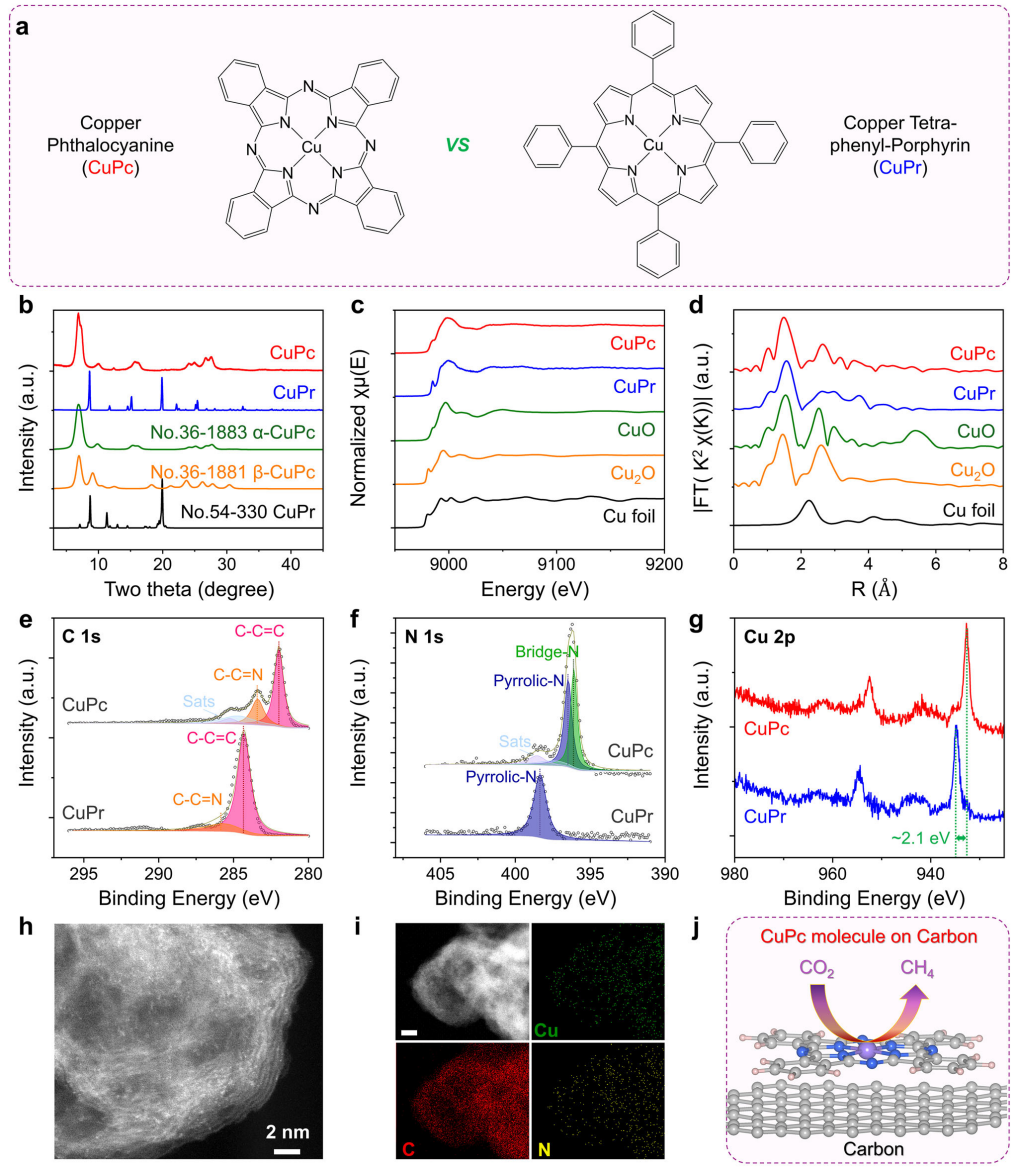
Structural characterization and property comparison of CuPc and CuPr. (a) Structural comparison of CuPc and CuPr. (b) XRD patterns of CuPc and CuPr compared with standard reference cards. (c, d) Cu K‐edge XANES (c) and EXAFS (d) spectra of CuPc and reference (CuPr, CuO, Cu_2_O, and Cu foil). (e‐g) XPS spectra of CuPc and CuPr in the C 1s (e), N 1s (f), and Cu 2p (g) regions. (h) TEM image of the CuPc/KB hybrid. (i) Elemental mappings of Cu, N, and C, scale bar: 5 nm. (j) Proposed reaction pathway for the conversion of CO_2_ to CH_4_ on the CuPc supported on carbon (CuPc/C) model.

Inspired by previous studies [13, 19, 24, 25, 26], we selected two representative Cu–N–C molecular catalysts, copper phthalocyanine (CuPc) and copper tetraphenylporphyrin (CuPr), as model systems to clarify how the macrocyclic structure affects electrocatalytic performance (Figure 1a). Both molecules possess a typical Cu–N_4_ coordination environment, in which the Cu center is coordinated by four nitrogen atoms in a planar configuration. In CuPc, the macrocyclic framework consists of four isoindole units linked by bridge nitrogen atoms, whereas CuPr contains four phenyl substituents at the meso positions of the porphyrin ring. These structural differences lead to distinct molecular frameworks and electronic properties.

X‐ray diffraction (XRD) analysis shows that the CuPc powder exhibits the characteristic α‐CuPc crystalline phase (PDF No. 36‐1883), whereas the diffraction peaks of CuPr match well with the standard pattern (PDF No. 54‐330). The standard reference cards were obtained from the database included in the Search‐Match software (Figure 1b). These results are consistent with the structural differences between the two molecular backbones.

To further probe the local electronic structures of CuPc and CuPr, X‐ray absorption fine structure (XAFS) measurements were performed. The X‐ray absorption near‐edge structure (XANES) spectra show that both CuPc and CuPr have absorption edge positions and near‐edge features more similar to CuO than to metallic Cu foil or Cu_2_O (Figure 1c). Since CuO, Cu_2_O, and Cu foil are commonly used as references for Cu^2+^, Cu^+^, and Cu^0^, respectively, these results suggest that the average Cu oxidation state in both CuPc and CuPr is close to +2. Moreover, the similar XANES line shapes suggest that the Cu centers in the two molecules have broadly comparable electronic states and local symmetry.

Extended X‐ray absorption fine structure (EXAFS) analysis was further carried out to examine the local coordination environment (Figure 1d). In the Fourier‐transformed EXAFS spectra, Cu foil exhibits a pronounced Cu–Cu coordination peak at R ≈ 2.1 Å, whereas Cu_2_O and CuO show Cu–O coordination peaks at R ≈ 1.6∼1.7 Å [9, 27]. In contrast, both CuPc and CuPr display a clear first‐shell peak at a distinctly different position, indicating that the Cu centers are coordinated by light elements rather than Cu neighbors. Combined with the molecular structures, this peak can be assigned to Cu–N coordination. The overall similarity of the spectra suggests comparable local coordination environments around the Cu centers. However, noticeable differences remain in the higher‐shell region from 2 to 4 Å, where CuPc shows more pronounced oscillations [9, 19, 27]. These differences may originate from enhanced multiple scattering, local structural distortion, and variations in the Cu–N bond‐length distribution caused by differences in the macrocyclic backbone.

To further examine their electronic structures, UV–vis absorption spectra were recorded for CuPc and CuPr. The CuPc solution appears blue, whereas the CuPr solution appears red, indicating distinct absorption behavior in the visible region. As shown in Figure S1, CuPc exhibits a prominent Q‐band absorption around 730 nm and another absorption feature near 280 nm in the ultraviolet region. This behavior arises from its more extended π‐conjugated system, which enables absorption in the red region and gives rise to the blue solution color. By contrast, CuPr shows a strong Soret‐band absorption around 400 nm, corresponding mainly to absorption in the blue region and thus a red solution color. These characteristic absorption features reflect the distinct electronic structures and π‐conjugation characteristics of the two molecules.

X‐ray photoelectron spectroscopy (XPS) measurements were then carried out using catalyst inks coated on single‐crystal silicon substrates. The survey spectra show that CuPc exhibits a much stronger N 1s signal than CuPr, indicating a higher nitrogen content in CuPc (Figures S2 and S3). Deconvolution of the C 1s spectra further shows that the relative area of the C–C═N‐related peak is significantly larger in CuPc, suggesting a greater abundance of C─N bonding motifs (Figure 1e). Analysis of the N 1s spectra reveals a distinct bridge‐N component in CuPc, whereas CuPr is dominated by pyrrolic‐N species (Figure 1f). In the Cu 2p spectra, the binding‐energy scale was calibrated using the Si 2p peak of the single‐crystal Si substrate at 99.4 eV (Figure S4), rather than the commonly used adventitious C 1s peak at 284.8 eV, because both CuPc and CuPr intrinsically contain carbon, making it difficult to distinguish the intrinsic carbon signals of the samples from adventitious carbon contamination. After calibration, the Cu 2p peak of CuPc shifts negatively by approximately 2.1 eV relative to that of CuPr (Figure 1g). This relative shift indicates a clear difference in electronic structure between the two molecules and suggests that the bridge‐N unit increases the electron density of the system, which may improve conductivity and benefit subsequent electrocatalytic reactions.

To examine the structural assembly of the catalyst, transmission electron microscopy (TEM) was performed. After loading onto Ketjen Black (KB), CuPc forms a thin crystalline layer on the carbon surface (Figure 1h), consistent with our previous observations [25, 28]. Elemental mapping further confirms the homogeneous distribution of Cu, N, and C, indicating that CuPc is highly dispersed on KB and forms a CuPc/KB hybrid structure (Figure 1i). Small‐angle X‐ray scattering (SAXS) was further used to compare pristine CuPc powder with the CuPc/KB hybrid (Figures S5 and S6). While the CuPc powder shows multiple characteristic scattering peaks, CuPc/KB differs from pure KB mainly at Q ≈ 10–12 nm^−1^. Together with the XRD results (Figure S7), in which no distinct CuPc diffraction peaks are observed for CuPc/KB, these findings further support the formation of a highly dispersed thin crystalline CuPc layer on the KB surface. Taken together, these results suggest that the Pc macrocyclic backbone modulates the electronic structure of the Cu center and interfacial charge‐transfer behavior, possibly through its extended π‐conjugated Pc ring and bridge‐N environment. These findings also indicate that the catalytically relevant structure is a CuPc/carbon hybrid interface rather than an isolated molecular catalyst in the conventional sense. Accordingly, a structural model of CuPc adsorbed on a carbon surface (CuPc/C) is proposed as the catalytically relevant structure for ECR (Figure 1j).

### 2.2 Electrochemical CO2 Reduction Performance of CuPc

Following our previously established procedure [24, 28, 29, 30], 300 mg of the CuPc/KB composite catalyst (20 wt%) was dispersed in 90 mL of solution and spray‐coated onto MFK‐A carbon paper to fabricate gas‐diffusion electrodes (GDEs). The catalyst loading was precisely controlled by adjusting the number of spray‐coating passes. After cutting and masking, the resulting GDEs were used as cathodes in a flow‐cell configuration for ECR tests (Scheme S1).

**FIGURE 2:**
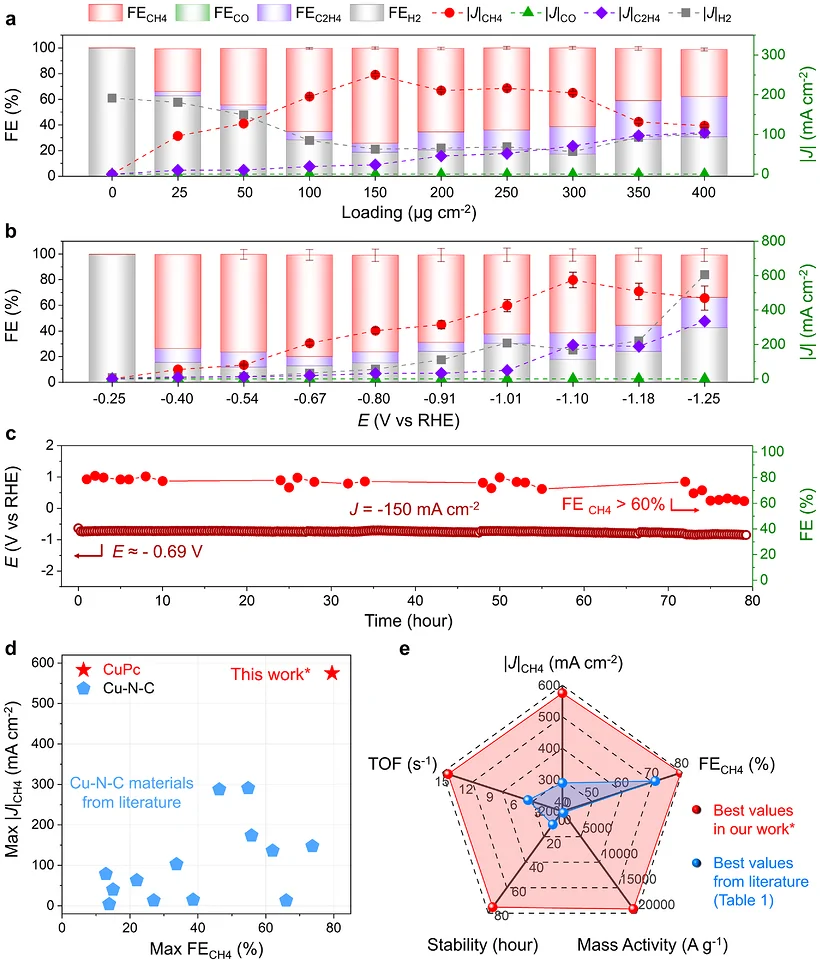
Electrocatalytic performance of CuPc/KB GDEs for ECR. (a) Faradaic efficiency (FE, left axis) and absolute current density (|*J*|, right axis) for electrodes with different catalyst loadings at a potential (*E*) of ‐0.91 V (vs. RHE). (b) FE (left axis) and |*J*| (right axis) as functions of *E* (vs. RHE) for electrodes with a loading of 150 µg cm^−2^ (6 passes). (c) Long‐term stability of the CuPc/KB GDE at a constant *J* of ‐150 mA cm^−2^, showing the variations in *E* (left axis) and FE (right axis) over time. (d,e) Comparison of maximum FE and |*J*| for CH_4_ in this work with other literature values (d), and radar plots showing best metrics from this work (red) versus the other literature (blue) (e).

We first investigated the effect of catalyst loading on ECR performance. After a single spray‐coating pass, the loading of CuPc/KB is approximately 25 µg cm^−2^, corresponding to a CuPc loading of about 5 µg cm^−2^, and CH_4_ can already be detected (Figure 2a and Figure S8). As the number of spraying passes increases, both the catalyst loading and the number of accessible active sites increase, leading to a clear enhancement in CH_4_ production. When the loading reaches approximately 150 µg cm^−2^ after six spray passes, the CH_4_ Faradaic efficiency (FE_CH4_) exceeds 70%. Upon further increasing the loading, the fraction of C_2_H_4_ gradually rises. This behavior is consistent with prior literature [9, 11, 14, 17, 27], suggesting that higher CuPc loadings may promote the formation of transient Cu cluster‐like species and thereby increase the probability of C─C coupling. Therefore, a catalyst loading of approximately 150 µg cm^−2^, obtained after six spray passes, was selected as the optimal condition for this study.

The ECR performance of CuPc/KB at the optimized loading was then evaluated under different applied potentials. The CuPc/KB catalyst exhibits a very low onset potential of −0.4 V versus RHE, at which nearly 60% CH_4_ selectivity is already achieved (Figure 2b). As the potential becomes more negative, the CH_4_ production rate increases rapidly and reaches a maximum CH_4_ partial current density (*J*_CH4_) of −575 mA cm^−2^ at −1.10 V versus RHE. Among the metrics reported, this value is the highest *J*_CH4_ reported so far for phthalocyanine‐based molecular catalyst systems (Table 1).

**TABLE 1:** Comparison of the key‐values in this work and selected literature.

Catalyst	pH	FE_CH4_ [%]	*J*_CH4_ [mA cm^−2^]	TOF [s^−1^]	MA [A g^−1^]	Stability @ *J* [hour @ mA cm^−2^]	Refs.
CuPc/KB	14.0	79.5	−575	14.3	19166.7	80 @ ‐150	This work*
CuPr‐4NH_2_	14.0	54.8	−290.5	0.28	290.5	∼9.5 @ ‐500	[21]
CuPr‐4Br	14.0	55.8	−173.6	0.501	173.6	∼11 @ ‐100	[19]
CuPr‐pristine	14.0	22	−62.9	0.12	62.9	∼11 @ ‐100	[19]
CuPr‐4OM	14.0	33.7	−102.7	0.237	102.7	∼11 @ ‐100	[19]
CuPr‐8OH	∼7.1	27	−13.2	4.3	52.8	—	[11]
CuPc‐Cl_4_/CNT	7.1	73.7	−147.4	—	139.1	10 @ ‐200	[13]
CuPc/CNTs	∼7.1	66	−13	0.39	216.7	—	[9]
Cu‐N‐C‐HKUST‐1	14.0	15	−40	—	71.4	∼8 @ ‐1.8 V	[14]
Cu‐N‐C‐MAF‐2ME	7.1	9.4	—	—	—	4 @ ‐1.3 V	[41]
Cu‐N‐C‐MAF‐2E	7.1	16.7	—	—	—	8 @ ‐1.3 V	[41]
Cu‐N‐C‐PzH	14.0	12.90	−78.5	—	78.5	∼4 @‐1.0 V	[20]
Cu‐N‐C‐PzI	14.0	46.30	−287.5	—	287.5	∼3.3 @‐1.0 V	[20]
Cu−N−C‐800	7.1	13.9	−3.83	—	3.83	—	[15]

According to the relationship: ***J*_partial_ = *J*_total_ × FE**, the high *J*_CH4_ arises from the synergistic combination of a large total current density (*J*_total_) and high selectivity. The high *J*_total_ value reflects the abundance of accessible active sites and the optimized gas–liquid–solid three‐phase interfacial structure, whereas the high FE mainly originates from the excellent intrinsic catalytic properties of the molecular active sites. Together, these factors account for the outstanding electrocatalytic performance of CuPc/KB.

In addition to its high current density, we further evaluated its long‐term stability. As shown in Figure 2c, under galvanostatic operation at −150 mA cm^−2^, the CuPc/KB GDE maintains a stable operating potential of approximately −0.69 V vs RHE over 80 h of continuous electrolysis, demonstrating excellent durability. The slight potential fluctuation during long‐term operation is mainly attributed to minor wetting effects in the GDE rather than catalyst deactivation. Post‐reaction XPS and XANES analysis show that the Cu‐N_4_ sites remain essentially unchanged before and after electrolysis (Figures S9 and S10), further confirming the structural stability of the CuPc active sites during CO_2_ reduction.

To benchmark the overall catalytic performance, we compiled representative phthalocyanine‐based electrocatalysts reported in recent years, and the key performance metrics are summarized in Table 1. For clearer comparison, the maximum *J*_CH4_ and FE_CH4_ values are highlighted in Figure 2d. The results show that the CuPc catalyst developed in this work outperforms all previously reported phthalocyanine‐based catalyst systems. In addition, Table 1 summarizes other important metrics, including turnover frequency (TOF) and mass activity (MA), which are further illustrated in the radar plot (Figure 2e). Notably, the mass activity of CuPc reaches 19,166.7 A g^−1^, approximately 66 times higher than the previously reported maximum value of 290.5 A g^−1^, indicating exceptionally high catalytic efficiency on a mass basis. Likewise, the TOF reaches 14.3 s^−1^, far exceeding the previously reported value of 4.3 s^−1^. Taken together, these results demonstrate that the CuPc catalyst simultaneously delivers high current density, high selectivity, and excellent stability, highlighting its strong potential for practical CO_2_‐to‐CH_4_ electrosynthesis.

### 2.3 Mechanistic Insights Into the Enhanced Activity of CuPc Over CuPr

**FIGURE 3:**
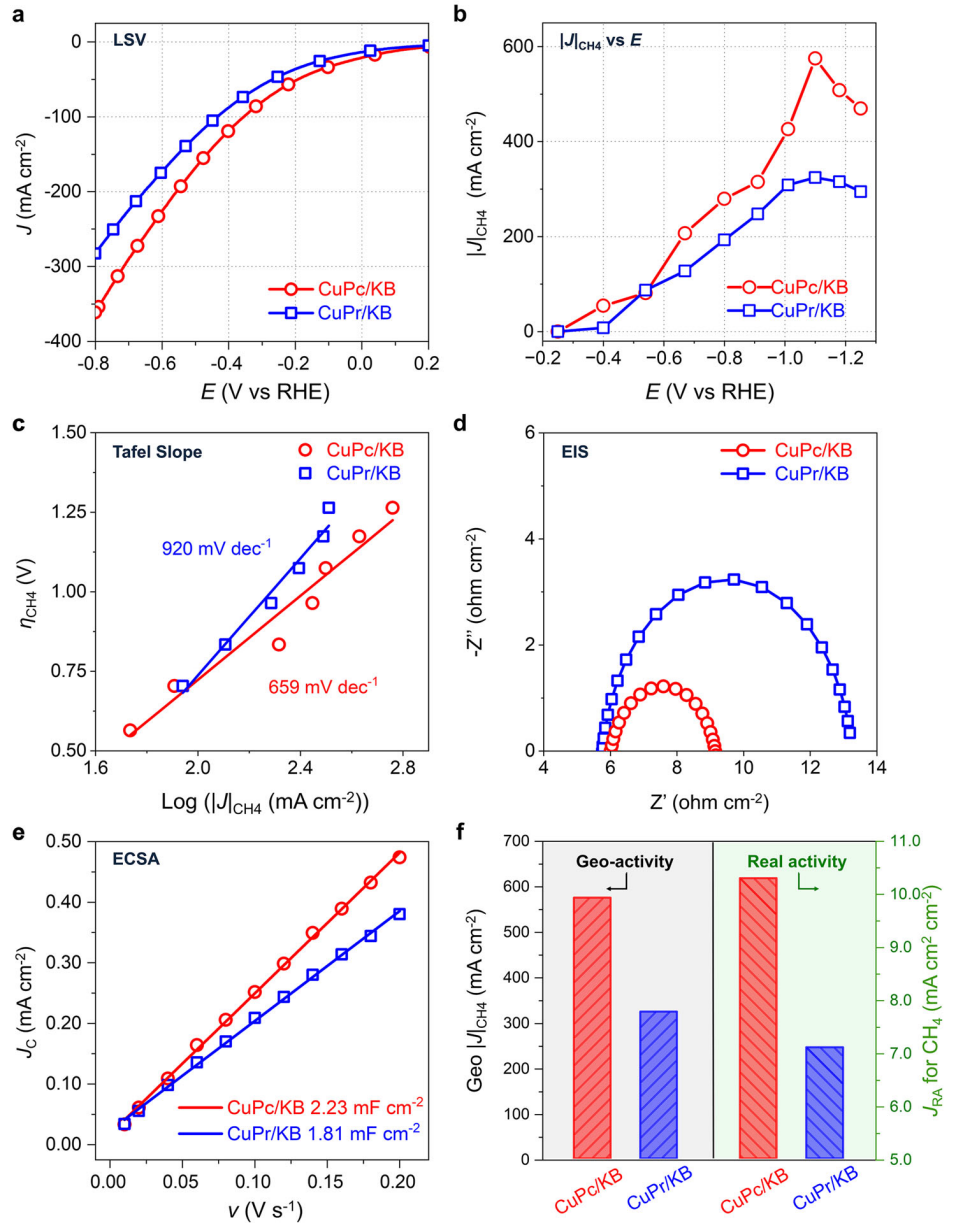
Mechanistic insights into the enhanced ECR performance of CuPc compared with CuPr. (a) Linear sweep voltammetry (LSV) curves of CuPc and CuPr GDEs. (b) Maximum |*J*|_CH4_ values of CuPc and CuPr GDEs. (c) Tafel plots and corresponding slopes for CuPc and CuPr GDEs. (d) Electrochemical impedance spectroscopy (EIS) spectra of CuPc and CuPr GDEs. (e) Double‐layer capacitance (C_dl_) of CuPc and CuPr GDEs. (f) Comparison of the maximum CH4 partial current density based on geometric area (left) and real intrinsic activity normalized by electrochemical surface area (ECSA) (right), demonstrating the superior per‐site activity of CuPc GDE than that of CuPr GDE.

To better understand the origin of the superior performance of CuPc, we carried out a systematic comparison with CuPr. We first examined their electrochemical behavior under CO_2_. As shown in Figure 3a, CuPc exhibits a substantially higher current density than CuPr at the same applied potential in the linear sweep voltammetry (LSV) curves, suggesting higher apparent catalytic activity. A direct comparison of CH_4_ production further shows that CuPc reaches a maximum CH_4_ partial current density of −575 mA cm^−2^, whereas CuPr delivers only −324 mA cm^−2^ (Figure 3b). In addition, the Tafel slope of CuPc is significantly smaller than that of CuPr (Figure 3c), suggesting more favorable apparent reaction kinetics for ECR. In general, a smaller Tafel slope indicates more facile charge‐transfer behavior during electrocatalysis. Electrochemical impedance spectroscopy (EIS) measurements further reveal that the charge‐transfer resistance of the CuPc‐based electrode is clearly lower than that of CuPr (Figure 3d), indicating more efficient interfacial electron transfer. Surface resistivity measurements using a four‐point probe further show different charge‐transport properties between CuPc and CuPr (Table S2). These results suggest that the phthalocyanine framework provides an advantage in modulating the electronic structure and interfacial charge‐transfer behavior.

To distinguish surface‐area effects from intrinsic catalytic activity, cyclic voltammetry (CV) was performed at different scan rates in the non‐Faradaic region (Figures S11 and S12) [31, 32, 33, 34], and the electrochemically active surface area (ECSA) was estimated from the double‐layer capacitance (*C*_dl_). Under the same catalyst loading condition (six spray passes), the *C*_dl_ values of CuPc and CuPr are 2.23 and 1.81 mF cm^−2^, corresponding to ECSA values of 55.7 and 45.3 cm^2^ cm^−2^, respectively (Figure 3e). Since KB contributes significantly to the measured surface area, these ECSA values are used here as relative indicators of the accessible electrode/electrolyte interface under comparable conditions, rather than as absolute active‐site densities. Under their respective optimal reaction conditions, the maximum |*J*|_CH4_ values of CuPc and CuPr are 575 and 324 mA cm^−2^, respectively. After ECSA normalization, the corresponding intrinsic current densities are 10.3 and 7.2 mA unit^−1^ (Figure 3f). These results indicate that the performance advantage of CuPc mainly arises from its higher intrinsic catalytic activity rather than from a simple difference in surface area.

**FIGURE 4:**
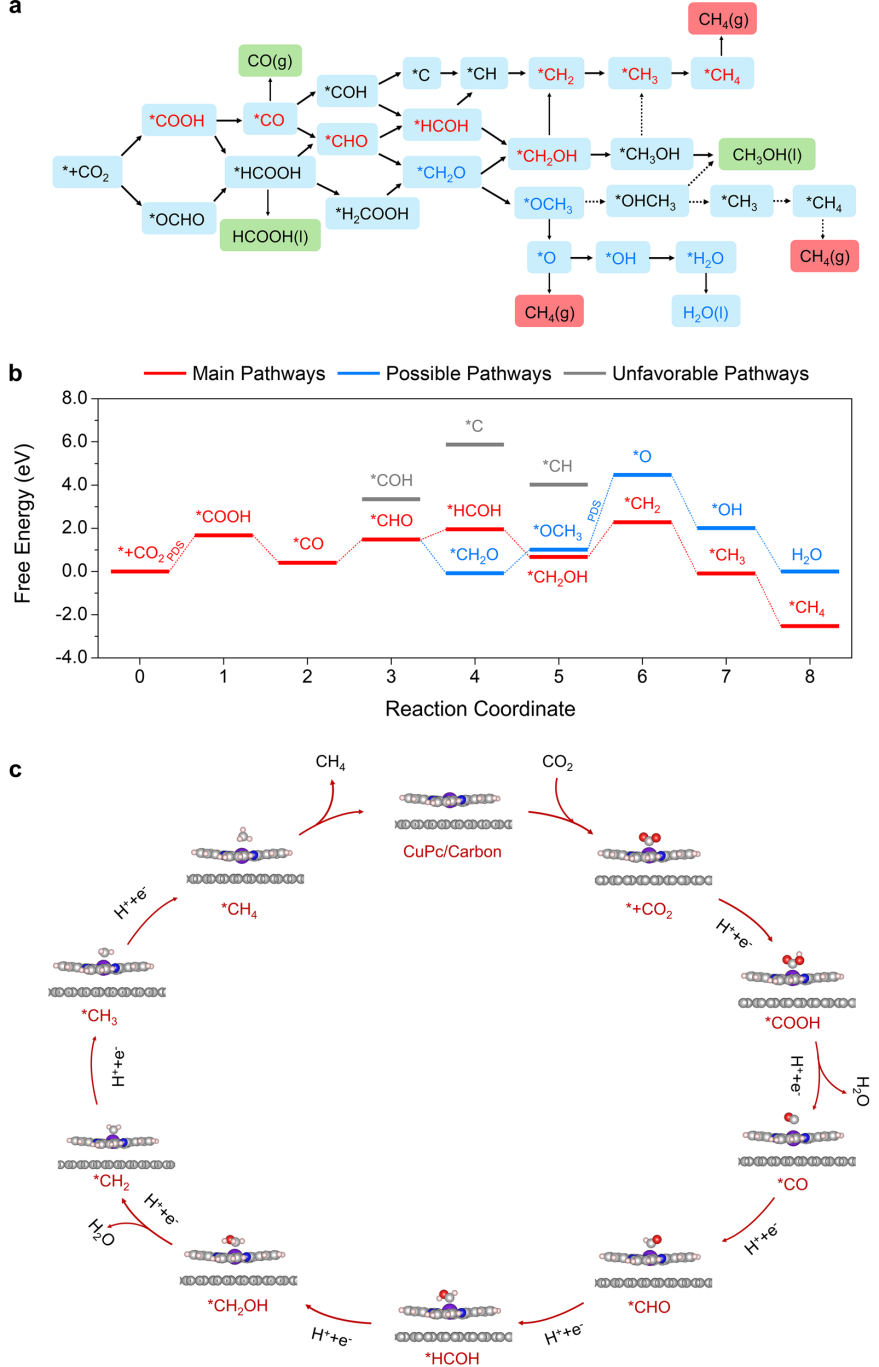
Electrocatalytic mechanism of the CuPc/C model for ECR. (a) Reaction pathways for CO_2_ electroreduction toward the target product CH_4_ (pink) and side products, including CO, HCOOH, CH_3_OH (green). (b) Free‐energy profiles of reaction intermediates on CuPc/C models for CO_2_‐to‐CH_4_ conversion, including the main pathway (red), the possible pathway (blue), and unfavorable pathways (gray). (c) Schematic illustration of the complete catalytic cycle for CO_2_‐to‐CH_4_ conversion on the CuPc/C model.

Based on previous studies [9, 35, 36, 37, 38, 39], we summarized a comprehensive CO_2_‐to‐CH_4_ reaction network involving multiple structurally related intermediates and competing products, including CO, HCOOH, and CH_3_OH (Figure 4a). This scheme provides a clearer overview of the possible reaction routes, which have not been fully discussed in previous studies. In constructing the reaction pathways, we considered previous experimental findings showing that formate/formic acid and methanol/methoxide are generally difficult to further reduce toward methane under typical ECR conditions [36]. Specifically, the –OH group in formic acid is acidic and can readily deprotonate in alkaline KOH electrolyte, making formate a relatively stable end product. Similarly, methanol and methoxide have been reported to be largely inactive toward further reduction to methane [9, 35, 36, 37, 38, 39].

It should be noted that the nature of the active sites in Cu–N–C molecular catalysts during ECR remains under discussion. Some previous studies have suggested that Cu nanoparticles or Cu clusters formed during ECR may act as the active species [9, 27], whereas others have reported that the molecular structures may remain stable under ECR conditions, with the Cu–N_4_ moiety or the entire molecular catalyst serving as the active unit [13, 19]. Our previous studies showed that M–Pc molecular catalysts, such as CoPc‐ and CoTAP‐based systems [24, 25, 29, 40], can remain stable during ECR. In addition, Yue et al. and Zhang et al. reported that CuPc and CuPr, respectively, could remain structurally stable during ECR [13, 19]. In the present work, post‐electrolysis XPS and XANES characterizations show no obvious formation of derived metallic Cu species (Figures S9 and S10). Therefore, we consider the molecular CuPc and CuPr catalysts, rather than reconstructed Cu clusters, as the main active units for ECR under our reaction conditions.

To gain deeper insight into the reaction mechanism, we combined experimental observations with DFT calculations based on CuPc molecules adsorbed on carbon, denoted as the CuPc/C model, to analyze the possible CO_2_‐to‐CH_4_ pathways. Based on the CuPc/C model, we systematically evaluated approximately 20 possible intermediates. The calculations indicate that CH_4_ formation on CuPc/C can proceed through a favorable pathway, while another competing pathway involving oxygenated intermediates is also possible (Figure 4b and Table S3). In detail, the most favorable CH_4_‐forming pathway follows the [*+CO_2_ → *COOH → *CO → *CHO → *HCOH → *CH_2_OH → *CH_2_ → *CH_3_ → *CH_4_] route, for which the [*+CO_2_ → *COOH] step is identified as the rate‐determining step (RDS) or potential‐determining step (PDS) with a free‐energy barrier of 1.68 eV [36, 38]. In addition, we also examined a possible competing oxygenated‐intermediate pathway, the [*+CO_2_ → *COOH → *CO → *CHO → *CH_2_O → *OCH_3_ → *O → *OH → *H_2_O] route. This pathway exhibits a much higher free‐energy charge of approximately 3.46 eV for the [*OCH_3_ → *O] step, suggesting that it is thermodynamically less favorable under standard computational hydrogen electrode (CHE) conditions, although it may become accessible under strongly cathodic potentials. To further understand the electronic interaction associated with the PDS, differential charge density analysis of the COOH‐adsorbed CuPc/C model reveals pronounced interfacial charge redistribution upon adsorption (Figure S13). Electron accumulation is mainly localized at the adsorbate–catalyst interface, accompanied by electron depletion around the Cu center and its coordination environment, suggesting that the Cu center facilitates electron transfer toward the adsorbed COOH intermediate. Notably, further Bader charge analysis shows only a marginal change of 0.006 |e| at the Cu center upon adsorption, suggesting that the specific N‐coordination environment primarily promotes local electron redistribution rather than substantially altering the charge state of Cu, thereby facilitating the interaction with key ECR intermediates.

According to the report by Zhang and co‐workers [19], CuPr follows a reported CH_4_‐forming pathway, namely [*+ CO_2_ → *COOH → *CO → *CHO → *OCH_2_ → *OCH_3_ → *OCH_4_], in which the [*COOH → *CO] step is the PDS with an energy charge of 1.83 eV [19]. The lower PDS free‐energy barrier of the CH_4_‐forming pathway on CuPc/C compared with that reported for CuPr suggests that CuPc/C provides a more favorable thermodynamic pathway for CH_4_ formation. The most favorable pathways are summarized in Figure 4c. Taken together, these results suggest that the macrocyclic backbone and its specific N‐coordination environment regulate the local electronic structure around the Cu center, promote interfacial electron redistribution, and modulate the adsorption energetics of key ECR intermediates, thereby influencing the preferred reaction pathway and CH_4_ selectivity during ECR.

## 3 Conclusions

In summary, we systematically compared two representative Cu–N–C molecular catalysts, CuPc and CuPr, for CO_2_‐to‐CH_4_ electroreduction and clarified the role of the macrocyclic backbone in CH_4_ production. CuPc exhibited markedly superior performance, achieving a maximum FE_CH4_ of 79.5%, a high *J*_CH4_ of −575 mA cm^−2^, a mass activity of 19,166.7 A g^−1^, and stable long‐term operation for over 80 h at a current density of −150 mA cm^−2^, outperforming previously reported phthalocyanine‐based catalysts. DFT calculations indicate that CuPc/C provides a more favorable CH_4_‐forming pathway than that reported for CuPr, with a lower free‐energy barrier for the potential‐determining step. Together with its higher ECSA‐normalized relative activity, these results suggest that CuPc possesses more favorable catalytic properties under comparable electrode conditions. This work shows that the phthalocyanine macrocyclic backbone can regulate reaction pathways and intermediate adsorption energetics, possibly through electronic modulation associated with its extended π‐conjugated Pc‐ring and bridge‐N environment. These findings provide mechanistic insight into the rational design of molecular catalysts for CO_2_‐to‐CH_4_ electroreduction.

## Author Contributions

T.L. conducted conceptualization, investigation, methodology, resources, formal analysis, and writing of the original draft. X.H. conducted formal analysis. S.Y. handled conceptualization and methodology. K.I., Y.H., Y.M., and S.O. participated in methodology, resources, and formal analysis. H.L. and H.Y. led conceptualization, resources, Writing – review & editing, and supervision. T.L., H.L., and H.Y. coordinated collaboration among all co‐authors and finalized the draft. All authors contributed to the revisions.

## Conflicts of Interest

The authors declare no conflicts of interest.

## Supporting information




**Supporting File**: smll75041‐sup‐0001‐SuppMat.docx.

## Data Availability

The data that support the findings of this study are available in the Supporting Information of this article.
