# Supplementary material for: Unraveling Structure‐Dependent Performance of Cu–N–C Molecular Catalysts for Electrocatalytic CO2 Methanation
Source: Small. 2026 Aug 2;22(50):e75041. doi: 10.1002/smll.75041 (PMC13548912; doi:10.1002/smll.75041)
Supplement: Supplementary file 1 [file SMLL-22-e75041-s001.docx]

The Supporting Information for

**Unraveling Structure-Dependent Performance of Cu–N–C Molecular Catalysts for Electrocatalytic CO_2_ Methanation**

Tengyi Liu,^†,*^ Xiaofan Hou,^†^ Songbo Ye,^†^ Kosuke Ishibashi, Yutaro Hirai, Yasutaka Matsuo, Shimpei Ono, Hao Li,^*^ and Hiroshi Yabu^*^

* Corresponding Authors:

T. Liu, Senior Assistant Professor, WPI-AIMR, Tohoku University, Sendai, Japan; Tel/Fax: +81-022-217-6341; Email: [liu.tengyi.e1@tohoku.ac.jp](mailto:liu.tengyi.e1@tohoku.ac.jp); Orcid: 0000-0003-3581-1842.

H. Li, Professor/Principal Investigator, WPI-AIMR, Tohoku University, Sendai, Japan; Tel/Fax: +81-022-217-6371; Email: [li.hao.b8@tohoku.ac.jp](mailto:li.hao.b8@tohoku.ac.jp); Orcid: 0000-0002-7577-1366.

H. Yabu, Professor/Principal Investigator, WPI-AIMR, Tohoku University, Sendai, Japan; Tel/Fax: +81-022-217-5996; Email: [hiroshi.yabu.d5@tohoku.ac.jp](mailto:hiroshi.yabu.d5@tohoku.ac.jp); Orcid: 0000-0002-1943-6790.

**[^†^]** These authors contributed equally to this work.

**Contents**

[**S-1. Experiment Section**	3](#_Toc234935638)

[**S-1.1. Materials and Regents**	3](#_Toc234935639)

[**S-1.2 Equipment and Devices**	3](#_Toc234935640)

[**S-1.3. Synthesis and Preparation**	4](#_Toc234935641)

[**S-1.3-1. Preparation of Carbon-Supported Cu-N-C Hybrid**	4](#_Toc234935642)

[**S-1.3-2. Preparation of CuPc/KB Electrodes**	5](#_Toc234935643)

[**S-1.4. Electrochemical CO_2_ Reduction (ECR) Test**	5](#_Toc234935644)

[**S-1.5. Qualitative and Quantitative Analysis of ECR Products**	6](#_Toc234935645)

[**S-1.6. Turnover Frequency and Mass Activity**	7](#_Toc234935646)

[**S-1.7. Computational Methods**	8](#_Toc234935647)

[**Scheme S1.** Laboratory electrochemical CO_2_ reduction (ECR) system	10](#_Toc234935648)

[**Table S1.** Chemical reactions and numbers of electrons transferred	11](#_Toc234935649)

[**Figure S1.** UV-Vis-NIR spectra	12](#_Toc234935650)

[**Figure S2.** Unprocessed XPS spectra of CuPc powder on a Si substrate	13](#_Toc234935651)

[**Figure S3.** Unprocessed XPS spectra of CuPr powder on a Si substrate	14](#_Toc234935652)

[**Figure S4.** XPS spectra in the Si 2p region before and after binding-energy correction	15](#_Toc234935653)

[**Figure S5.** Typical 2D SAXS patterns (Q-zone)	16](#_Toc234935654)

[**Figure S6.** Typical 1D-integrated SAXS patterns	17](#_Toc234935655)

[**Figure S7.** Typical XRD patterns	18](#_Toc234935656)

[**Table S2.** Surface resistivity of blank carbon paper, CuPc and CuPr GDEs	19](#_Toc234935657)

[**Figure S8.** Typical GC-TCD spectra of the gaseous products obtained from CuPc/KB electrode	20](#_Toc234935658)

[**Figure S9.** Cu 2p spectra of the CuPc/KB electrode	21](#_Toc234935659)

[**Figure S10.** Cu K-edge XANES of the CuPc/KB electrode	22](#_Toc234935660)

[**Table S3.** Reaction intermediates on CuPc/C models for CO_2_-to-CH_4_ conversion	23](#_Toc234935661)

[**Figure S11.** ECSA measurement of CuPc/KB electrodes	25](#_Toc234935662)

[**Figure S12.** ECSA measurement of CuPr/KB electrodes.	26](#_Toc234935663)

[**Figure S13.** Differential charge density of the *COOH-adsorbed CuPc/C model.	27](#_Toc234935664)

[**Reference in the Supporting Information**	28](#_Toc234935665)

# **S-1. Experiment Section**

## **S-1.1. Materials and Regents**

CuPc and CuPr were synthesized or supplied by Azul Energy Corporation, each with a purity higher than 99.9%. Ketjen-Black carbon black (EC-600JD) was sourced from Fuel Cell Store. 2-Propanol (IPA) and dimethyl sulfoxide (DMSO) were obtained from Fuji-Wako, each with a purity above 99.5%. Nafion dispersion solution (20 wt%) was purchased from Sigma-Aldrich. The carbon paper gas diffusion layers used for electrochemical CO_2_ reduction tests were acquired from Mitsubishi Chemical Corporation (PYROFIL-GDL MFK-A, 0.21 mm), while those (ABOUND-GDL) for long-term durability tests were supplied by Abound Energy Corporation. All gases, including carbon dioxide (CO_2_), carbon monoxide (CO), methane (CH_4_), ethylene (C_2_H_4_), and hydrogen (H_2_), were purchased from Taiyo Nippon Sanso Company, each with a purity over 99.9%. Ultrapure water (18.2 MΩ) was obtained through a purification system. All reagents were used as received without further purification.

**S-1.2 Equipment and Devices**
 The following equipment was used in this study. A ball-mill (FRITSCH, Planetary Mono Mill Pulverisette 6) ground materials into fine powder, while a sim-coat system (Sono-Tek) applied uniform catalyst films onto substrates. Gas chromatography with flame ionization detection (GC-FID, Shimadzu GC-2014) analyzed gaseous products, and an electrochemical workstation (Princeton Applied Research, Versa-STAT 4) conducted electrochemical measurements, including CO_2_ reduction tests. An analytical balance (A&D, BM-20) ensured precise mass measurements, and an oven (Espec, ST-120) was used for controlled drying. An ultrasonic machine (Asone, ASU-2M) dispersed inks, and gas flow meters (FCON, C2005-CO_2_-003) monitored gas flows. Ultrapure water was generated using an ELGA (LA-758 Purelab system) purification system. X-ray photoelectron spectroscopy (XPS, Ulvac-PHI 5000 Versa Probe II) analyzed surface composition, X-ray diffraction (XRD, RIGAKU Smart-Lab) determined crystalline structures, and X-ray absorption fine structure (XAFS, SPring-8 & NanoTerasu) spectroscopy provided local electronic insights. The raw data were analyzed using the ATHENA software packages. Fourier transformations of the k^3^-weighted EXAFS spectra were conducted for all samples over a k-space range of 3–10 Å^-1^. For the resulting data, analyzing the R-space range from 0 to 6 Å (or up to 10 Å if needed) was deemed sufficient in this work. A scanning electron microscope (SEM, JEOL JSM-7800F) and field emission electron probe microanalysis (FE-EPMA, JEOL JXA-8530F) were used for imaging and elemental analysis. The surface resistivity was measured using a Mitsubishi Chemical four-point probe system (MCP-T610) equipped with a PSP probe

**S-1.3. Synthesis and Preparation**

**S-1.3-1. Preparation of Carbon-Supported Cu-N-C Hybrid**

In this work, Cu-N-C hybrid, including CuPc/KB and CuPr/KB catalysts were prepared using a method further refined from our previous studies ^[1–3]^. For instance, a 20 wt% CuPc/KB catalyst was synthesized by first dissolving 200 mg of CuPc in 150 mL of DMSO in a round-bottom flask, followed by the addition of 800 mg of Ketjen Black (KB), maintaining a total solid mass of 1000 mg. This fixed solid-to-solvent ratio was kept constant across all samples. The resulting mixture was sonicated in an ice-water bath for 30 minutes to promote the adsorption of CuPc onto the KB surface. Sonication was carried out using a probe sonicator set to an amplitude of 1 and a 30% pulse cycle, as higher amplitudes could result in the dissolution of CuPc rather than its adsorption. After adsorption, the mixture was filtered, and the solid was washed three times with methanol, then dried at a moderate temperature (e.g., 35 °C) in a vacuum oven for 24 hours to preserve catalytic activity. The dried product was ground using a mortar, weighed, and transferred to a ball-mill autoclave. The ball-milling solvent consisted of DMSO (9 mL), IPA (9 mL), H_2_O (4.5 mL) and Nafion (20 wt%, 0.1 mL per 50 mg catalyst). Ball milling was conducted at 400 rpm for 5 minutes, followed by reverse rotation for another 5 minutes, repeated for a total of 30 minutes to reduce carbon agglomeration. After milling, the ball-mill autoclave was rinsed with a solvent mixture containing DMSO (9 mL), IPA (45 mL), and H_2_O (13.5 mL), bringing the total solvent volume to 90 mL. The resulting mixture was then ultrasonically dispersed for at least 30 minutes to obtain a uniform catalyst ink.

**S-1.3-2. Preparation of CuPc/KB Electrodes**

The resulting ink was spray-coated onto carbon paper to fabricate the CuPc/KB hybrid electrode. Different catalyst loadings were achieved by adjusting the ink concentration and the number of spray passes. A slim-coating system (Flex-Coat Max, Sono-Tek) was used for spray-coating. Specifically, 30 mL of ink was loaded into a tempered glass container, and computer-controlled feeding delivered the ink to an ultrasonic nozzle (factory-original component, Sono-Tek), with dry, oil-free air at 551.6 kPa (80 PSI) propelling the ink droplets onto the carbon substrate. The substrate was masked using a stainless-steel square stencil (6 × 6 cm^2^) to define the catalyst deposition area and was heated on a hot plate at 70 °C to facilitate solvent evaporation and promote uniform spray-deposition/growth of the catalyst across the carbon substrate surface. Catalyst loading was tuned by either increasing the ink concentration or the number of spray coatings. However, an ink concentration of >1000 mg/90 mL might lead to nozzle clogging. Therefore, a standard ink formulation of 300 mg CuPc/KB dispersed in 90 mL of solution—corresponding to 60 mg of CuPc—was adopted to ensure stable and consistent spray performance. In this study, 10 spray passes were identified as the optimal condition for uniform electrode fabrication, the loading of CuPc/KB is about 0.25 mg cm^-2^ (the pure CuPc loading is 0.05 mg cm^-2^).

**S-1.4. Electrochemical CO_2_ Reduction (ECR) Test**

The prepared electrode was cut and used as the working electrode in a custom three-electrode electrolyzer, with Hg/HgO (1.0 M KOH) as the reference electrode and Pt wire as the counter electrode, the detailed components and main equipment are reported previously. The 1.0 M KOH solution served as the electrolyte. The carbon-based gas diffusion electrode (GDE) was masked with a tape ring (0.5 cm² hole) and used as the cathode, with CO_2_ fed from the back of the GDE, and the flow controlled by a flow meter (e.g., 15 sccm). This setup optimizes the solid-liquid-gas interface and prevents electrode flooding. The anode and cathode chambers were separated by a Nafion-117 membrane, which was chosen to avoid the transfer of ECR products into the anode, as this could cause errors, even though an anion exchange membrane may offer higher exchange ratios.^[4]^ After connecting the setup to the electrochemical workstation, gas products were collected in a gas bag, and their volume was measured using a flow meter. The gases were analyzed by GC-FID and selectivity and partial current density were obtained (details in the next section). The applied potentials were adjusted to optimize faradaic efficiency and current density for CO_2_ reduction. The potential vs. reference was converted to the reversible hydrogen electrode (RHE) using the equation:

*E* (vs. RHE) = *E* (vs. Ref.) + 0.0592 × pH + *E*₀ (Ref.) - *iR*

Where *E* (vs. Ref.) is the applied potential, *E*₀ (Ref.) is the standard potential of the reference electrode (0.105 V for Hg/HgO in 1.0 M KOH),^[5]^ and *iR* is the ohmic drop.

For standard ECR tests, electrolysis was performed on an MFK-A GDE for about 900 seconds using 25 mL KOH electrolyte in both the cathode and anode chambers. For long-term durability tests, liquid pumps were used to refresh the anolyte and catholyte, and ABOUND-GDL was employed to prevent flooding.

**S-1.5. Qualitative and Quantitative Analysis of ECR Products**

In this work, we use gas chromatography with a flame ionization detector (GC-FID) to analyze the ECR products.^[2,3,6]^ Gases displayed different peak positions in real-time based on molar mass, with lighter gases appearing earlier and heavier gases later in the intensity vs. time pattern. The main ECR products included H_2_, CH_4_, CO, and C_2_H_4_. The first three gases were detected using a thermal conductivity detector (TCD), while C_2_H_4_ was detected only by FID, likely due to FID's sensitivity to hydrocarbons. Following the principles of GC, H_2_, CH_4_, and CO eluted in sequence with retention times around 0.7, 3.0, and 5.9 minutes, respectively. Slight variations in peak times occurred due to carrier gas flow rates but did not affect the overall peak order, enabling reliable qualitative analysis.

Peak area in the intensity vs. time graph is proportional to gas concentration. Calibration lines were created for four gases by preparing standard mixtures. For example, a 20% CH_4_ standard was made by mixing 80 mL of CO_2_ with 20 mL of CH_4_ in a sample bag and analyzing it with GC-FID. Each sample was measured three times for accuracy. Calibration curves showed R² values over 0.99, indicating high reliability.

During ECR electrolysis, gas products were collected in sample bags, and total volume was recorded. Gas content and peak areas were analyzed with GC-FID, and gas ratios were determined using the calibration curves. The detailed ECR system and electrolyzer are shown in **Scheme S1**. From this, the molar quantities of each gas were calculated. For example, CH_4_ moles (n_CH4_) were determined from peak areas. Using the CO_2_-to-CH_4_ reaction stoichiometry, the electron transfer for CH_4_ production was calculated with Q_CH4_ = n_CH4_ × F × N_CH4_, where F is Faraday's constant (96485 C/mol), and N_CH4_ is the number of electrons transferred (N_CH4_ = 8). The chemical reactions and corresponding numbers of electrons transferred for each reaction are shown in **Table S1**.^[7,8]^

The total charge (Q_total_) was determined by Q_total_ = I × t, where I is the current recorded by the electrochemical workstation, and t is the relative time. Faradaic efficiency for CH_4_ (FE_CH4_) was calculated as FE_CH4_ = Q_CH4_ / Q_total_. Finally, the total current density (*J*_total_) was calculated by dividing the current by the electrode surface area (0.5 cm²), and the partial current density for CH_4_ (*J*_CH4_) was determined using *J*_CH4_ = *J*_total_ × FE_CH4_.

**S-1.6. Turnover Frequency and Mass Activity**

In this work, the TOF was calculated based on the CH_4_ partial current density and the amount of CuPc or CuPr loaded on the electrode. The number of active sites was estimated from the molecular loading, assuming one Cu–N_4_ center per CuPc or CuPr molecule.

For CH_4_ formation, eight electrons are required per CH_4_ molecule. Therefore, the TOF was calculated using the following equation:

TOF = *J*_CH4_ × A / (8 × F × nCu)

where *J*_CH4_ is the CH_4_ partial current density, A is the geometric electrode area, F is the Faraday constant, and nCu is the mole number of Cu molecular sites calculated from the CuPc or CuPr loading.

In this work, the CuPc/KB loading was approximately 0.25 mg cm^-2^, corresponding to a pure CuPc loading of approximately 0.05 mg cm^-2^. The mass activity was calculated by normalizing the CH_4_ partial current to the mass of CuPc or CuPr loaded on the electrode.

**S-1.7. Computational Methods**
 In this study, density functional theory (DFT) calculations were carried out using the Vienna Ab initio Simulation Package (VASP) to determine the adsorption energies and free-energy changes of key CO_2_RR intermediates. The exchange–correlation interactions were described using the revised Perdew–Burke–Ernzerhof (RPBE) functional within the generalized gradient approximation (GGA).^[9–11]^ The Kohn–Sham equations were solved using a plane-wave basis set with a kinetic energy cutoff of 520 eV, and the projector augmented-wave (PAW) method was employed to describe the electron–ion interactions.^[11,12]^

The electronic and ionic relaxations were considered converged when the energy difference was below 10^-5^ eV and the residual forces were less than 0.05 eV Å^-1^, respectively. The Brillouin zone was sampled using a Monkhorst–Pack k-point grid, ensuring that k × a exceeded 15 Å. Spin polarization was considered throughout all calculations. To avoid interactions between periodic images, a vacuum region of at least 15 Å was applied along the surface-normal direction.

The reaction free energies were evaluated using the computational hydrogen electrode (CHE) model. Unless otherwise specified, the free-energy diagrams were constructed at 0 V versus the reversible hydrogen electrode (RHE). The Gibbs free-energy change of each elementary proton–electron transfer step was calculated according to:

ΔG = ΔE + ΔE_ZPE_ − TΔS + ΔG_U_ + ΔG_pH_

where ΔE is the reaction energy obtained from DFT calculations, ΔE_ZPE_ is the zero-point energy correction, T is the temperature, and ΔS is the entropy change. The temperature was set to 298.15 K. The potential correction is given by ΔG_U_ = −neU, where n is the number of transferred electrons, e is the elementary charge, and U is the applied potential versus RHE. Since the free-energy profiles were evaluated at 0 V vs. RHE, ΔG_U_ is zero under this condition. The pH correction term was treated within the RHE scale and was therefore not explicitly included in the free-energy diagrams at 0 V vs. RHE.

Zero-point energy and entropy corrections were included for the adsorbed intermediates and gas-phase molecules. The vibrational frequencies of adsorbed intermediates were calculated to obtain the zero-point energy and vibrational entropy contributions, while the entropy values of gas-phase molecules were taken from standard thermodynamic data. For adsorbed intermediates, translational and rotational entropy contributions were neglected, and only vibrational entropy was considered.

For CH_4_ formation, the free-energy changes of possible ECR intermediates were calculated on the CuPc/C model. The potential-determining step (PDS) was identified as the elementary step with the largest uphill free-energy change along the reaction pathway. We note that these calculations are based on thermodynamic free-energy profiles within the CHE model; therefore, the term PDS is used instead of rate-determining step unless kinetic barriers or transition states are explicitly calculated.

To verify the reliability of the calculated values, we rechecked the free-energy profiles and compared the key energy barriers with previously reported CuPc- and CuPr-based systems. The calculated free-energy barriers are within a reasonable range and support the conclusion that CuPc/C provides a more favorable thermodynamic pathway for CH_4_ formation than the reported CuPr pathway.

**Scheme S1.** Laboratory electrochemical CO_2_ reduction (ECR) system: (**a**) Main components and equipment of the laboratory ECR system; (**b**) Schematic diagram, and (**c**) Actual image of the custom-made three-electrode electrolyzer.

**Table S1.** Chemical reactions and numbers of electrons transferred for each reaction.^[7,8]^

| **Product** | **N** | **Half Reaction (pH =14)** | ***E*_0_ (vs. RHE)** |
| --- | --- | --- | --- |
| **H_2_** | 2 | 2H_2_O (l) + 2e^−^ → H_2_ (g) + 2OH^−^ | 0 |
| **CO** | 2 | CO_2_ (g) + H_2_O (l) + 2e^−^ → CO (g) + 2OH^−^ | -0.106 |
| **CH_4_** | 8 | CO_2_ (g) + 6H_2_O (l) + 8e^−^ → CH_4_ (g) + 8OH^−^ | 0.164 |
| **C_2_H_4_** | 12 | 2CO_2_ (g) + 8H_2_O (l) + 12e^−^ → C_2_H_4_ (g) + 12OH^−^ | 0.074 |

**Figure S1.** UV-Vis-NIR spectra of CuPc and CuPr.

**Note:** CuPc and CuPr solutions in DMSO appear blue and red, respectively. The solutions were prepared at a concentration of 2 mg catalyst in 5 mL DMSO. A quartz cuvette containing 285 μL of DMSO was first used as the background reference, followed by the addition of 15 μL of catalyst ink for UV–Vis measurement. The CuPc-containing solution shows a prominent Q-band absorption around 730 nm and another feature near 280 nm, consistent with its extended π-conjugated structure. In contrast, the CuPr-containing solution shows a strong Soret-band absorption around 400 nm. These features reflect the distinct electronic structures and π-conjugation characteristics of the two molecules.

**Figure S2.** Unprocessed XPS spectra of CuPc powder on a Si substrate, as obtained directly from the XPS instrument: (**a**) Survey spectrum, (**b**) C 1s, (**c**) N 1s, (**d**) Cu 2p, and (**e**) Si 2p spectra.

**Note:** Compared with CuPr (**Figure S3**), CuPc shows a significantly stronger N signal in the survey spectrum, consistent with its molecular structure. In addition, the high-resolution C 1s, N 1s, and Cu 2p spectra show binding-energy shifts of approximately 2.1 eV, whereas the Si 2p peak remains unchanged (**Figure S4**), indicating that these shifts originate from structural differences rather than instrumental or calibration errors.

**Figure S3.** Unprocessed XPS spectra of CuPr powder on a Si substrate, as obtained directly from the XPS instrument: (**a**) Survey spectrum, (**b**) C 1s, (**c**) N 1s, (**d**) Cu 2p, and (**e**) Si 2p spectra.

**Figure S4.** XPS spectra in the Si 2p region before and after binding-energy correction.

**Figure S5.** Typical 2D SAXS patterns (Q-zone) of (**a**) Blank KB, (**b**) CuPc, and (**c**) 20 wt% CuPc/KB hybrid powders.

**Figure S6.** Typical 1D-integrated SAXS patterns of blank KB, CuPc, and 20 wt% CuPc/KB hybrid powders.

**Figure S7.** Typical XRD patterns of blank KB, and CuPc/KB hybrid powders.

**Table S2.** Surface resistivity of blank carbon paper, CuPc and CuPr GDEs.

| **Surface Resistivity**  **(Ω unit^-1^)** | **Blank GDE** | **CuPc GDE** | **CuPr GDE** |
| --- | --- | --- | --- |
| 1 | 0.7818 | 0.7864 | 0.8683 |
| 2 | 0.7310 | 0.8280 | 0.8574 |
| 3 | 0.7446 | 0.8103 | 0.8343 |
| 4 | 0.7963 | 0.8043 | 0.8475 |
| 5 | 0.7741 | 0.7926 | 0.8488 |
| 6 | 0.7876 | 0.7877 | 0.8198 |
| 7 | 0.7863 | 0.7609 | 0.8316 |
| 8 | 0.7723 | 0.7668 | 0.8276 |
| 9 | 0.7526 | 0.8149 | 0.8722 |
| 10 | 0.7414 | 0.8126 | 0.8776 |
| Average | 0.7668 | 0.7965 | 0.8485 |

**Note:** The surface resistivity of the GDEs was measured using a Mitsubishi Chemical four-point probe system (MCP-T610) equipped with a PSP probe. The GDE samples were cut into circular pieces with a diameter of 28 mm for measurement. To keep the samples flat and prevent movement during testing, each GDE was placed in a 100 mL beaker, and the probe was gently brought into contact with the surface to avoid damaging the catalyst film. The thickness of the carbon paper was approximately 0.23 mm. The Pc-based catalyst films were coated on the GDEs following our previously reported method (Ref. 3), and the film thickness was estimated to be several micrometers. The obtained surface resistivity values are used only for relative comparison of charge-transport properties among the GDEs. The CuPc-based GDE shows a slightly lower surface resistivity than the CuPr-based GDE, suggesting relatively more favorable charge transport, which is consistent with the electrochemical trends observed in this study.

**Figure S8.** Typical GC-TCD spectra of the gaseous products obtained from CuPc/KB electrode for CO_2_ reduction electrolysis.

**Figure S9.** Cu 2p spectra of the CuPc/KB electrode before and after long-term CO_2_ reduction electrolysis.

**Figure S10.** Cu K-edge XANES of the CuPc/KB electrode before and after long-term CO_2_ reduction electrolysis.

**Table S3.** Reaction intermediates on CuPc/C models for CO_2_-to-CH_4_ conversion.

| **Structure** | **Intermediate** | **Structure** | **Intermediate** |
| --- | --- | --- | --- |
|  | Pristine CuPc/C |  | *+CO_2_ |
|  | *COOH |  | *CO |
|  | *OCHO |  | *HCOOH |
|  | *CHO |  | *COH |
|  | *HCOH |  | *H_2_CO |
|  | *C |  | *H_3_CO |
|  | *CH |  | *H_2_COH |
|  | *CH_2_ |  | *H_3_COH |
|  | *CH_3_ |  | *CH_4_ |

**Figure S11.** ECSA measurement of CuPc/KB electrodes. (**a**) Cyclic voltammetry (CV) curves of CuPc/KB electrodes in 1 M KOH solution at various scan rates, ranging from -0.05 to 0.05 V vs. Hg/HgO. (**b**) Double-layer capacitance (*C*_dl_) derived from fitting the capacitance current density (*j*_C_) against the scan rate (*v*) for CuPc/KB/CP electrodes.

**Note:** CV scans were performed on CuPc/KB to calculate the *C*_dl_ at varying scan rates, allowing for the determination of the ECSA.^[4,15]^The results indicate that the *C*_dl_ value for CuPc/KB is 2.23 mF cm^-2^, corresponding to an ECSA of 55.75 cm^2^ cm^-2^. These findings clearly demonstrate that the incorporation of KB significantly increases the ECSA. While ECSA primarily reflects the solid-liquid interface, previous studies suggest that a higher ECSA enhances the three-phase boundary, which facilitates the exposure of more active sites for CO_2_RR.

**Figure S12.** ECSA measurement of CuPr/KB electrodes. (**a**) Cyclic voltammetry (CV) curves of CuPr/KB electrodes in 1 M KOH solution at various scan rates, ranging from -0.05 to 0.05 V vs. Hg/HgO. (**b**) Double-layer capacitance (*C*_dl_) derived from fitting the capacitance current density (*j*_C_) against the scan rate (*v*) for CuPr/KB/CP electrodes.

**Note:** The results indicate that the *C*_dl_ value for CuPr/KB is 1.81 mF cm^-2^, corresponding to an ECSA of 45.25 cm^2^ cm^-2^.

**Figure S13.** Differential charge density of the *COOH-adsorbed CuPc/C model.

**Note:** Yellow and cyan regions represent electron accumulation and depletion, respectively. Pronounced interfacial charge redistribution is observed upon *COOH adsorption, with electron accumulation mainly localized around the adsorbate–catalyst interface and electron depletion around the Cu center and its coordination environment. This result suggests that the Cu center facilitates electron transfer toward the adsorbed *COOH intermediate. Bader charge analysis further shows only a marginal charge variation of 0.006 |e| at the Cu center upon adsorption, suggesting that the specific N-coordination environment primarily promotes local electron redistribution rather than substantially altering the charge state of Cu.

**Reference in the Supporting Information:**

[1]	H. Abe, Y. Hirai, S. Ikeda, Y. Matsuo, H. Matsuyama, J. Nakamura, T. Matsue, H. Yabu, *NPG Asia Mater.* **2019**, *11*, 57.

[2]	T. Liu, D. Zhang, Y. Chu, K. Ohashi, Y. Hirai, K. Ito, K. Ishibashi, Y. Matsuo, J. Yoshida, S. Ono, K. Kamiya, H. Li, H. Yabu, *Appl. Catal. B Environ.* **2026**, *381*, 125852.

[3]	T. Liu, D. Zhang, Y. Hirai, K. Ito, K. Ishibashi, N. Todoroki, Y. Matsuo, J. Yoshida, S. Ono, H. Li, H. Yabu, *Adv. Sci.* **2025**, *12*, 202501459.

[4]	T. Liu, K. Ohashi, K. Nagita, T. Harada, S. Nakanishi, K. Kamiya, *Small* **2022**, *18*, 2205323.

[5]	K. Kawashima, R. A. Márquez, Y. J. Son, C. Guo, R. R. Vaidyula, L. A. Smith, C. E. Chukwuneke, C. B. Mullins, *ACS Catal.* **2023**, *13*, 1893.

[6]	T. Liu, X. Hou, D. Zhang, Y. Hirai, K. Ishibashi, Y. Matsuo, J. Yoshida, S. Ono, H. Li, H. Yabu, *Small* **2025**, *21*, e07824.

[7]	Z. Yue, C. Ou, N. Ding, L. Tao, J. Zhao, J. Chen, *ChemCatChem* **2020**, *12*, 6103.

[8]	T. Liu, H. Yabu, *EcoEnergy* **2024**, *2*, 419.

[9]	B. Hammer, L. B. Hansen, J. K. Nørskov, *Phys. Rev. B - Condens. Matter Mater. Phys.* **1999**, *59*, 7413.

[10]	J. Y. Jung, J. H. Park, Y. J. Jeong, K. H. Yang, N. K. Choi, S. H. Kim, W. J. Kim, *Korean J. Physiol. Pharmacol.* **2006**, *10*, 289.

[11]	K. Li, L. Luo, Y. Zhang, W. Li, Y. Hou, *ACS Appl. Mater. Interfaces* **2018**, *10*, 41525.

[12]	A. Allouche, *J. Comput. Chem.* **2012**, *32*, 174.

[13]	K. Chan, C. Tsai, H. A. Hansen, J. K. Nørskov, *ChemCatChem* **2014**, *6*, 1899.

[14]	W. Yang, Z. Jia, B. Zhou, L. Chen, X. Ding, L. Jiao, H. Zheng, Z. Gao, Q. Wang, H. Li, *ACS Catal.* **2023**, *13*, 9695.

[15]	T. Liu, P. Diao, Z. Lin, H. Wang, *Nano Energy* **2020**, *74*, 104787.
